# Feline calicivirus- and murine norovirus-induced COX-2/PGE_2_ signaling pathway has proviral effects

**DOI:** 10.1371/journal.pone.0200726

**Published:** 2018-07-18

**Authors:** Mia Madel Alfajaro, Eun-Hyo Cho, Jun-Gyu Park, Ji-Yun Kim, Mahmoud Soliman, Yeong-Bin Baek, Mun-Il Kang, Sang-Ik Park, Kyoung-Oh Cho

**Affiliations:** Laboratory of Veterinary Pathology, College of Veterinary Medicine, Chonnam National University, Gwangju, Republic of Korea; Tulane University, UNITED STATES

## Abstract

Cyclooxygenases (COXs)/prostaglandin E_2_ (PGE_2_) signaling pathways are known to modulate a variety of homeostatic processes and are involved in various pathophysiological conditions. COXs/PGE_2_ signaling pathways have also been demonstrated to have proviral or antiviral effects, which appeared different even in the same virus family. A porcine sapovirus Cowden strain, a member of genus *Sapoviru*s within the *Caliciviridae* family, induces strong COX-2/PGE_2_ but transient COX-1/PGE_2_ signaling to enhance virus replication. However, whether infections of other viruses in the different genera activate COXs/PGE_2_ signaling, and thus affect the replication of viruses, remains unknown. In the present study, infections of cells with the feline calicivirus (FCV) F9 strain in the genus *Vesivirus* and murine norovirus (MNV) CW-1 strain in the genus *Norovirus* only activated the COX-2/PGE_2_ signaling in a time-dependent manner. Treatment with pharmacological inhibitors or transfection of small interfering RNAs (siRNAs) against COX-2 enzyme significantly reduced the production of PGE_2_ as well as FCV and MNV replications. The inhibitory effects of these pharmacological inhibitors against COX-2 enzyme on the replication of both viruses were restored by the addition of PGE_2_. Silencing of COX-1 via siRNAs and inhibition of COX-1 via an inhibitor also decrease the production of PGE_2_ and replication of both viruses, which can be attributed to the inhibition COX-1/PGE_2_ signaling pathway. These data indicate that the COX-2/PGE_2_ signaling pathway has proviral effects for the replication of FCV and MNV, and pharmacological inhibitors against these enzymes serve as potential therapeutic candidates for treating FCV and MNV infections.

## Introduction

The *Caliciviridae* family is composed of small, non-enveloped, icosahedral viruses that possess a single-stranded, positive-sense RNA genome of 7–8 kb in size [1]. This family is comprised of five established genera, *Lagovirus*, *Nebovirus*, *Norovirus*, *Sapovirus*, and *Vesivirus* [2], with six additional unclassified new genera tentatively named *Recovirus* [3], *Bavovirus* [4, 5] *Nacovirus* [5–7], *Salovirus* [8], *Sanovirus* [9], and *Valovirus* [10]. The members in this family are important pathogens in both medical and veterinary fields [1]. For example, feline calicivirus (FCV) belonging to the genus *Vesivirus* causes acute, self-lining oral and upper respiratory tract disease in cats [11]. Moreover, virulent, systemic FCV mutant strains causing severe systemic diseases with edematous and ulcerative skin lesions, jaundice, and a high mortality of up to 67% have recently been found in the US and EU [12–14]. Porcine sapovirus (PSaV), and bovine norovirus and nebovirus cause widespread acute gastroenteritis in piglets and calves, respectively [15–17]. In the medical field, human norovirus and human sapovirus, especially the former, is the leading cause of gastroenteritis in humans, accounting for ~200,000 deaths per annum in children <5 [18–19].

FCV vaccines are available for cats [20]; however, there is a limit on its efficacy because of low or no preventive effect against FCVs with different antigenicity and short-lived immunity [13, 20]. A lack of a robust and reproducible *in vitro* cultivation system for human noroviruses (HNoVs) has not been developed until recently, thereby hindering the development of effective interventions [21]. Therefore, our understanding of the HNoV life cycle was largely based on those surrogate viruses specifically the murine noroviruses (MNVs) [22]. Therapeutic candidates for FCV and MNV may be utilized for various FCV strains and HNoVs [13].

The cyclooxygenase (COX) enzymes, which convert arachidonic acid into prostaglandins (PGs), orchestrate a variety of homeostatic processes and participate in various pathophysiological conditions such as inflammation and immune responses [23–28]. Currently three isoforms of COXs have been identified, with COX-1 and COX-2 being the most studied [29]. COX-1 functions as a housekeeping isoform of COX and is expressed constitutively to perform functions such as control of renal blood flow, platelet aggregation, and provide protection against stomach ulcers [29]. COX-2 is activated in response to different extracellular or intracellular stimuli, which can lead to the accumulation of PGs including PGE_2_ [30]. PGE_2_ is commonly involved in cellular immunity and inflammation events, and serves as strong regulators of cell–cell interaction, cytokine production, antigen presentation, cell differentiation and survival, apoptosis, and cell migration [30].

Viral infections generally impose immunological pressure on their hosts that in turn can hinder their successful replication [31]. To evade this, viruses devise strategies to subvert cellular pathways for their own benefit such as via COXs/prostaglandin E_2_ (PGE_2_) signaling, which participates in the modulation of the host response to infection and the life cycle of several viruses [32–33]. Representatives from at least eleven different virus families are known to activate COXs/PGE_2_ signaling or to enhance the expression levels of PGE_2_: *Caliciviridae* [34], *Arteriviridae* [35], *Herpesviridae* [36], *Rhabdoviridae* [37], *Retroviridae* [38], *Picornaviridae* [39–40], *Flaviviridae* [41], *Orthomyxoviridae* [42–43], *Adenoviridae* [44], and *Paramyxoviridae* [45].

Some viruses including vesicular stomatitis virus (VSV) [37, 46], enterovirus 71 (EV71) [39–40, 47]), dengue virus [41], hepatitis C virus (HCV) [48–49], Japanese encephalitis virus [46], influenza virus [42, 50], cytomegalovirus (CMV) [36, 51], herpes simplex virus type 1 (HSV-1) [52], and HSV-6 [53] are known to use the COX-2/PGE_2_ signaling pathway for their benefit rather than COX-1/PGE_2_ signaling pathway [33]. However, porcine reproductive and respiratory syndrome (PRRS) arterivirus has been shown to hijack the COX-1/PGE_2_ signaling pathway and induce fever [35], and pseudorabies herpesvirus has been shown to use both COX-1 and COX-2/PGE_2_ pathways for its successful replication [54]. In contrast, *in vitro* and/or *in vivo* treatment of PGE_2_ reduces replication of hepatitis B virus [43, 55], adenoviruses [44], parainfluenza virus [56], and measles virus [45], suggesting an antiviral function of PGE_2_ to these viruses. Moreover, the COXs/ PGE_2_ signaling pathway or their final product PGE_2_ has been shown to facilitate or inhibit the replication of viruses in the same family. For example, in the *Retroviridae* family, the COXs/PGE_2_ signaling pathway and/or PGE_2_ enhances the replication of bovine leukemia virus [38], as well as human T-cell leukemia virus-I [57] and III [58], but inhibits human immunodeficiency virus-1 (HIV-1) in monocyte-derived macrophage [59]. It should be noted that treatment of PGE_2_ enhances the replication of HIV-1 in the CD4+ T-cell line MT-4 [60], indicating also that depending on the cells, the role of COXs/PGE_2_ signaling pathways is different in the same viral infection.

Previously, we reported that infection of PSaV strain Cowden induced strong COX-2/PGE_2_ signaling with only a transient COX-1/PGE_2_ signal during late stage infection [34]. Pharmacological inhibitors or siRNAs against COX-1 and COX-2 significantly reduced PSaV replication, which was restored by the addition of PGE_2_, indicating that COXs/PGE_2_ acts as a proviral signal [34]. However, the role of COXs/PGE_2_ signaling is unknown for other members within the *Caliciviridae* family. This prompts us to investigate whether other members of caliciviruses, in particular the FCV and MNV strains, use the COX-2/PGE_2_ signaling pathway in the regulation of their own replication. Here, we demonstrated that COX-2 expression was induced during FCV and MNV infections, thereby causing a surge in PGE_2_ levels. Furthermore, pharmacological inhibitors and siRNAs against COX-1 and COX-2 enzymes significantly reduced PGE_2_ production as well as MNV and FCV replication, which could be restored by the addition of PGE_2_. These results suggest that COX-2/PGE_2_ pathway has proviral effects for FCV and MNV replication.

## Materials and methods

### Cells and viruses

RAW264.7 mouse macrophage and Crandell Rees feline kidney (CRFK) cells were purchased from the American Type Culture Collection (ATCC, Manassas, VI, USA) and were routinely grown in Dulbecco’s modified Eagle’s minimal essential medium (DMEM) supplemented with 10% fetal bovine serum (FBS), 100 U/ml penicillin, and 100 μg/ml streptomycin. The FCV F9 strain was obtained from ATCC and was propagated in CRFK cells. The MNV strain CW-1 was a kind gift from Dr. H.W. Virgin, Washington University School of Medicine, USA. Cesium chloride (CsCl) density gradient ultracentrifugation was performed to purify each mass-cultured strain as described previously [61–62]. The viral titer for MNV and FCV strains was determined by median tissue culture infectious dose (TCID_50_) in units per milliliter and immunofluorescence assay, respectively, as described below.

### Chemicals and antibodies

NS-398 and SC-560 were purchased from Cayman Chemical (Ann Arbor, MI, USA). Indomethacin and dimethyl sulfoxide (DMSO) were from Sigma-Aldrich (St. Louis, MO, USA). COX-1 and COX-2 siRNAs, which were comprised of three targets for each gene, and scrambled siRNA were purchased from Santa Cruz Biotechnology, Inc. (Dallas, TX, USA). Synthetic PGE_2_ was from Tocris Bioscience (Ellisville, MO, USA). Polyclonal antibody against rabbit COX-2 and monoclonal antibody against mouse COX-1 were acquired from Abcam (Cambridge, MA, USA). Mouse monoclonal antibody against FCV capsid protein was from Santa Cruz Biotechnology, Inc. The rabbit polyclonal MNV VPg antibody was a kind gift from Dr. I. Goodfellow, Cambridge University, UK. The secondary antibodies used were horseradish peroxidase-conjugated goat immunoglobulin against rabbit IgG (Cell Signaling, Beverly, MA, USA) and mouse IgG (Santa Cruz), and fluorescein isothiocyanae (FITC)-conjugated goat immunoglobulin against rabbit IgG and mouse IgG (Jackson Immuno Research Laboratory, West Grove, PA, USA).

### Cytotoxicity assay

The cytotoxicity test for the different chemicals used was carried out by a 3-(4,5-dimethylthiazol-2-yl)-2,5-diphenyl tetrazolium bromide (MTT) assay as described previously [34, 63]. Briefly, monolayers of RAW264.7 and CRFK cells grown in 96-well plates were incubated for 24 h with different concentrations of chemicals. After removal of the medium and washing twice with phosphate buffered saline (PBS, pH 7.4), 200 μl of MTT solution was added in each well and incubated for 4 h at 37°C in a CO_2_ incubator. Next, 150 μl of DMSO was added to each well and incubated for 10 min at room temperature. Optical density (OD) was determined in an enzyme-linked immunoabsorbent assay (ELISA) reader at 570 nm. Calculations for the percent cell viability was determine using the following formula: [(OD_sample_-OD _blank_)/ (OD_control_-OD_blank_)] × 100. Nontoxic concentrations of chemicals were utilized in this study.

### Treatment of cells with inhibitors

The inhibitors and chemicals used in this study were diluted in DMSO to a stock concentration of 10 mM and were subsequently diluted in media to make working solutions. Monolayers of RAW264.7 and CRFK cells grown in 6- or 12-well plates were treated with chemicals or inhibitors as described previously [34]: mock-treatment, pre-treatment, post-treatment, and pre-post-treatment. Briefly, confluent cells pretreated with various concentrations of the inhibitors for 24 h were infected with MNV (MOI, 1 TCID_50_/ml) or FCV (MOI, 1 FFU/ml) strains for the pre-treatment groups. For the post-treatment groups, cells were adsorbed with MNV (MOI, 1 TCID_50_/ml) or FCV (MOI, 1 FFU/ml) strains and then treated with different concentrations of inhibitors. For the pre-post-treatment groups, cells were pretreated with different concentrations of inhibitors for 24 h, adsorbed with MNV (MOI, 1 TCID_50_/ml) or FCV (MOI, 1 FFU/ml) strains, and treated again with the same concentration of inhibitors described above.

### Plasmid constructs

The cDNA from a partial part of the polymerase of the FCV F9 strain and a part of the N-terminal of the open reading frame (ORF) 1 of the MNV strain were amplified by conventional PCR using the primers listed in Table 1. The DNA fragments were gel purified using the Wizard® SV Gel and PCR Clean-Up system (Promega, Fitchburg, Wisconsin, USA) and ligated to pGEM-Teasy vector (Promega). Samples were transformed in homemade DH5α and positive colonies were picked by white and blue screening. The selected colonies were grown in LB-medium containing ampicillin. The plasmids were purified using Hybrid-Q Plasmid Rapidprep kit (GeneAll, Songpa-gu, Seoul, South Korea) and concentrations were determined by spectrophotometry (BioPhotometer plus, Eppendorf, Hamburg, Germany). The plasmids for FCV and MNV strains were named pTA-FCV and pTA-MNV-1, respectively.

**Table 1:** Oligonucleotides used in this study.

**Target gene**	**Sequence (5’-3’)**	**Region (nt)**	**Size (bp)**	**Reference**	**Accession no.**
MNV-1 (ORF1) *(RT-PCR)*	F: ATGAGGATGGCAACGCCATC R: ATGATCTCTATCTTCGGGGA	6–25 996–1015	1015	This study	DQ285629.1
FCV (RdRp) *(RT-PCR)*	F: CCGCTGTCCAAAATCTCTCA R: TCAGTAATCAATTCCCTTAA	3901–3920 4661–4680	779	This study	M86379.1
MNV-1 (ORF1) *(qPCR)*	F: GTGCGCAACACAGAGAAACG R: GCAGGAAGCTCAGCCCG	39–58 160–177	139	Taube et al., 2009	DQ285629.1
FCV (RdRp) *(qPCR)*	F: ACATTTCCTCGGAAACCTCT R: GGAGAAGGTTAGTGAAGGGA	4041–4060 4261–4280	239	This study	M86379.1
Murine COX-1	F: TTACCCTGGAGATGACGGGT R: GGTTTTCGTGGCTTGGCATT	1895–1914 2011–2030	136	This study	NM_008969
Murine COX-2	F: CTTCGGGAGCACAACAGAGT R: CACCTGAGCGGTTACCACTT	1067–1086 1202–1221	155	This study	NM_011198.4
Feline COX-1	F: GCAGTTGAGCGGTTACTTCC R: CGGGAGTACAGCTACGAGC	1134–1153 1282–1300	167	This study	XM_006939439
Feline COX-2	F: AAACACTCGGGAACTTCGCA R: CTTGCTGTTCCAACCCATGC	32–51 188–207	176	This study	EF036473
Murine beta-actin	F: TATAAAACCCGGCGGCGCA R: CTTTGCAGCTCCTTCGTTGC	1–19 64–83	83	This study	NM_007393
Feline beta actin	F: TCCTGGGTATGGAGTCCTGT R: TCTACGCTAACACGGTGCTG	609–628 690–709	101	This study	AB051104

### RNA extraction

Mock- or virus-infected, mock- or inhibitor-treated, or siRNA-transfected cells were washed thrice with cold PBS, scraped, and collected in clean microtubes. Harvested samples were centrifuged at 2,469 × g for 10 min, and then total RNA was extracted using the PureLink RNA minikit (Ambion Life Technologies, Carlsbard, CA, USA) following the manufacturer’s instructions. To quantify the genome copy numbers of FCV or MNV strains, the cells treated with inhibitors, infected with each strain, or transfected with each siRNA described above were freeze-thawed three times, and cell debris were removed by centrifugation at 2,469 × g at 10 min at 4°C. Samples were immediately processed or stored at -80°C until use. Total RNA was isolated from supernatants using a Viral RNA extraction kit (Bionner, Daejeon, South Korea) following the manufacturer’s instructions. Concentrations of extracted RNAs were calculated by spectrophotometry at 260 nm using the BioPhotometer plus (Eppendorf).

### Quantitative real-time PCR

A one-step real-time RT-PCR (qRT-PCR) assay with primer pairs specific to the polymerase of FCV and ORF1 of MNV was performed as described previously [61, 64–66]. The primer pairs used are listed in Table 1. Briefly, total RNA was isolated as described above from harvested samples that were subjected to three times freeze-thaw cycles. All reactions were carried out using a Corbett Research Rotor-Gene Real-time Amplification system (Corbett Research, Mortlake, Australia) and SensiFast™ SYBR® Lo-ROX One Step Kit (Bioline, London, UK). Next, 25 μl qRT-PCR reactions were prepared, which were composed of 5 μl RNA template, 10 μl SensiFast one-step mixture, 1 μl of forward and reverse primers (final concentration of each primer: 10 pmole), 0.2 μl reverse transcriptase, 0.4 μl of RiboSafe RNase inhibitor, and 7.4 μl of RNase free water. The amplification profile for FCV was as follows: reverse transcription at 45°C for 10 min, polymerase activation at 95°C for 2 min, and 45 cycles of amplification consisting of denaturation at 94°C for 10 s, primer annealing at 55°C for 20 s, and extension at 72°C for 10 s. For MNV-1, the reverse transcription was carried out at 45°C for 10 min, followed by polymerase activation at 95°C for 2 min, an initial denaturation at 95°C for 5 min, and 45 cycles of amplification consisting of denaturation at 94°C for 15 s, primer annealing at 57°C for 30 s, and extension at 72°C for 20 s. The copy numbers of the FCV and MNV-1 genes were calculated using 10-fold dilutions of a known concentration of *in vitro* transcribed complementary RNA using the pTA-FCV and pTA-MNV-1 plasmids to make the standard curve. Quantifications of RNA from samples were calculated using the Rotorgene 3000® (Corbett Research, Mortlake, Australia).

To determine the expression levels of COX genes in mock-, virus-, chemical-, or siRNA-treated samples, cells were harvested and total RNA were extracted as described above. RNA concentrations were determined, and cDNA was prepared using 1 μg of RNA and reverse transcribed using random hexamers (Roche, Basel, Switzerland). The specific primer pairs for qPCR were designed based on the published sequences for COX-1 and COX-2 as listed in Table 1. Reactions were carried out in a total volume of 25 μl containing 10 pmole of forward and reverse primers, cDNA, and TOPreal qPCR 2X Premix (Enzynomics, Daejon, South Korea). The amplification of COX-1 and COX-2 were as follows: initial denaturation at 95°C for 5 min, followed by 40 cycles of denaturation at 95°C for 10 s, primer annealing at 57°C for 20 s, and extension at 72°C for 10 s. The relative expressions of COX-1 and COX-2 were calculated via 2^-ΔΔCT^ using the Rotorgene 3000® software (Corbett Research, Mortlake, Australia) as described previously [34]. To normalize the samples, the β actin gene (Table 1) was quantified.

### Detection of PGE_2_ in cell culture supernatant by ELISA

Levels of PGE_2_ were determined via the PGE_2_ EIA kit (Cayman Chemical, Ann, Arbor, MI, USA) following the manufacturer’s instructions for clarified supernatants collected in different time points from the cells mock-infected or infected with each virus, mock-treated or treated with each chemical, or scrambled siRNA-transfected or transfected with siRNAs against COX-1 or COX-2. Using an ELISA reader, the absorbance was read at 405 nm and concentrations of PGE_2_ were calculated in comparison to a standard curve.

### TCID_50_ assay

The median tissue culture infectious dose (TCID_50_) of MNV and FCV strains in the samples obtained from the above experiments were determined as described previously [34]. Briefly, 10-fold serial dilutions of clarified supernatants were prepared. Confluent RAW264.7 and CRFK cells grown on 96-well plates were inoculated with 200 μl of each diluted sample and then incubated at 37°C in a 5% CO_2_ incubator. After 3–5 days postinfection, viral titers were calculated using the Reed and Muench method [67] and expressed as TCID_50_/ml.

### Silencing of COX-1 and COX-2 genes by transfection of siRNA

The siRNAs targeting COX-1 and COX-2 genes or scrambled siRNA were transfected to confluent CRFK and RAW 264.7 cells grown on 6- or 24-well plates using Lipofectamine 3000® reagent (Invitrogen, Carlsbad, CA, USA) according to the manufacturer’s instructions [34]. Cell were then mock-infected or infected with FCV (MOI, 1 FFU/ml) and MNV (MOI, 1 TCID_50_/ml) strains. Cells were subjected to qPCR, TCID_50_/ml, and Western blot analyses at the appropriate time points.

### Cell lysate preparation and Western blot analysis

Cells from the above experiments were washed, harvested, and lysed at different time points using a cell extraction buffer (Invitrogen) supplemented with protease and phosphatase inhibitors (Roche). Denatured cell lysates were resolved in sodium dodecyl sulfate (SDS) polyacrylamide gels and then transferred to nitrocellulose blotting membranes (Amersham Protran, GE Healthcare Life Science, Germany). Membranes were immunoblotted with primary antibodies against COX-1, COX-2, glyceraldehyde 3-phosphate dehydrogenase (GAPDH), MNV VPg, or FCV capsid. Secondary antibodies specific for rabbit or mouse IgG were added for 1 h at room temperature and were developed using an enhanced chemiluminescence reaction kit (DoGen, Nowon-gu, Seoul, South Korea). Images were captured on the Davinch-Western imaging system (Young Ltd., Kang-Nam, Seoul, South Korea). Samples were normalized to the corresponding GAPDH level in the same samples. The intensity of COX-1 and COX-2 proteins relative to GAPDH was determined by densitometric analysis.

### Immunofluorescence assay

The viral titer for FCV F9 strain was determined by immunofluorescence assays as described previously [12, 13]. Briefly, confluent CRFK cells grown on 96-well plates were inoculated with 200 μl of each diluted sample and then incubated at 37°C in a 5% CO_2_ incubator. After 4 hpi, cells were fixed with cold acetone. Cells were washed with PBS containing new fetal bovine serum (PBS-FBS) before incubating with anti-FCV capsid (1:100 dilution) overnight at 4°C. The cells were washed three times with cold PBS, and then FITC-conjugated goat anti-mouse IgG (Molecular Probes, Eugene, OR, USA) secondary antibody was added and incubated for 1 h at room temperature. Afterwards, slides were washed three times with cold PBS and mounted with 60% glycerol in PBS (pH 8.0). FCV titer was calculated using the Reed and Muench method [67] and expressed as fluorescence focus unit (FFU)/ml.

Infection inhibitory effects of each inhibitor or siRNA were determined by immunofluorescence assays as described previously [68]. Briefly, cells grown on 8-well chamber glass slides were treated with various inhibitors or transfected with siRNAs before or after infection of FCV (1 FFU/ml) or MNV (MOI, 1 TCID_50_/ml) strains as mentioned above. Then, cells were washed three times with cold PBS before being fixed with 4% formaldehyde in PBS. Cells were permeabilized with 0.02% Triton X-100, incubated at room temperature for 10 min, washed with PBS containing new fetal bovine serum (PBS-FBS) before incubating with anti-MNV VPg (1:100 dilution) or anti-FCV capsid (1:100 dilution) overnight at 4°C. The cells were washed three times with cold PBS, and then anti-rabbit or anti-mouse Alexa Fluor 488 (Molecular Probes, Eugene, OR, USA) secondary antibodies were added. Afterwards, slides were mounted with SlowFade Gold antifade reagent (Life Technologies, Eugene, OR, USA) containing DAPI (4’,6-diamidino-2-phenylindole) solution for nuclear staining and was visualize using the LSM 510 confocal microscope and analyzed using LSM software (Carl Zeiss, Oberkochen, Germany).

## Results

### FCV and MNV infections induce COX-2 expression that leads to the production of PGE_2_

**Fig 1:**
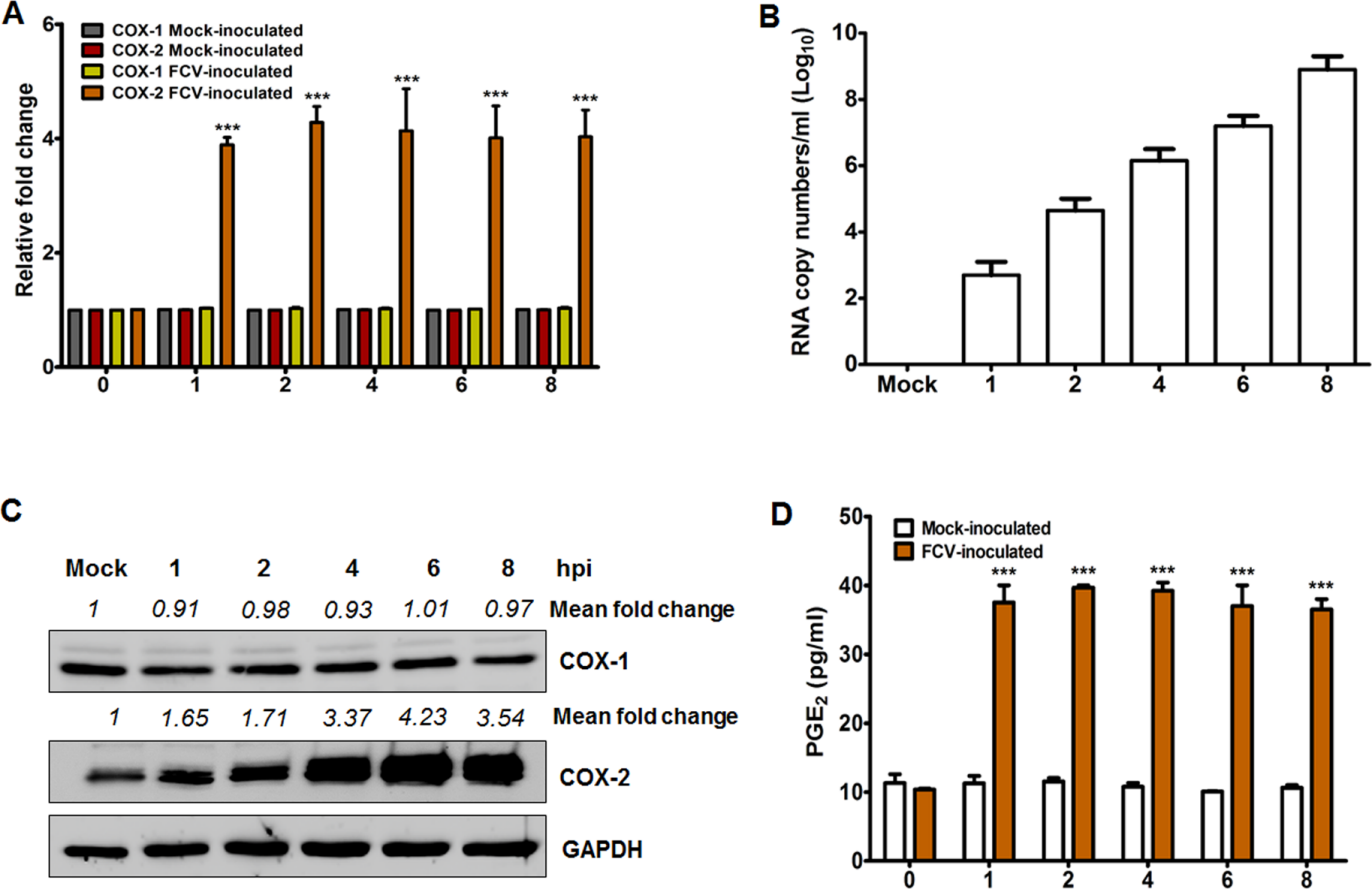
Feline calicivirus (FCV) infection induces COX-2 mRNA and protein expression leading to the production of PGE_2_. (A and B) The mRNA expression levels of COX-1 and COX-2 genes (A), and viral RNA level (B) in CRFK cells infected with or without FCV (MOI, 1 FFU/cell) at the indicated time points were determined by quantitative real time PCR. For COX-1 and COX-2, mRNA levels were normalized to β-actin mRNA and are illustrated as a fold induction against that of the mock-infected cells. (C) Monolayers of CRFK cells were infected with or without FCV (MOI, 1 TCID_50_/ml) for the indicated time points, and the levels of the COX-1, COX-2, and GAPDH proteins were analyzed by Western blot analysis. GAPDH was used as a loading control. The intensity of each target protein relative to GAPDH was determined by densitometric analysis and is indicated above each lane. (D) PGE_2_ levels in supernatants harvested from cells infected with or without FCV (MOI, 1 FFU/cell) at the indicated time points were analyzed by ELISA. The levels of PGE_2_ in supernatants were compared between mock-and FCV-infected groups. All data shown were from three independent experiments and are presented as means and standard errors of mean. Statistical differences were evaluated by one-way analysis of variance. *p < 0.05; **p < 0.001; ***p < 0.0001.

**Fig 2:**
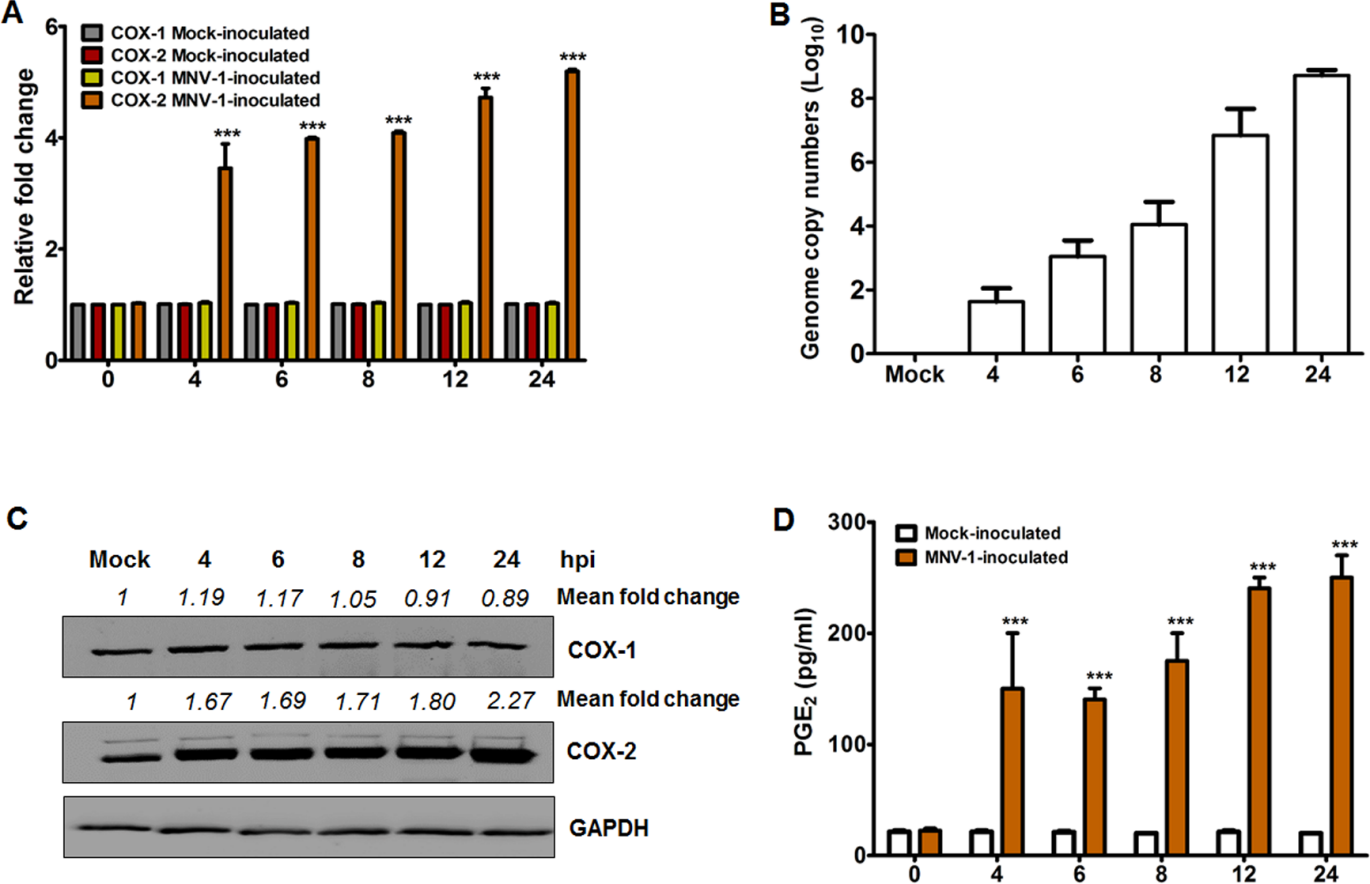
COX-2 mRNA and protein levels increase upon murine norovirus (MNV) infection with subsequent production of PGE_2_. (A and B) Cultured RAW264.7 cells were mock-infected or infected with MNV (MOI, 1 TCID_50_/ml) for the indicated time points, and the expression of COX-1 and COX-2 mRNAs, and MNV viral RNA were determined by quantitative real time PCR. The mRNA expression levels of COX-1 and COX-2 were normalized to β-actin mRNA and are presented as a fold induction as compared with the mock-infected cells. (C) Monolayers of RAW264.7 cells were infected with or without MNV (MOI, 1 TCID_50_/ml) for the indicated time points, and the levels of the COX-1, COX-2, and GAPDH proteins were analyzed by Western blot analysis. GAPDH was used as a loading control. The intensity of each target protein relative to GAPDH was determined by densitometric analysis and is indicated above each lane. (D) Cell culture supernatants harvested from the cells infected with or without MNV at the indicated time points were checked for the presence of PGE_2_ by ELISA. The levels of PGE_2_ in the supernatants were compared between mock-and MNV-infected groups. The presented data are depicted as means and standard errors of the mean from three different experiments. Statistical analyses were performed using one-way analysis of variance. ***p < 0.0001.

We previously reported that infection of LLC-PK cells with PSaV strain Cowden strongly activated the COX-2/PGE_2_ signaling pathway but transiently induced COX-1/PGE_2_ signaling pathway during the late stage of infection [34]; hence, we examined whether both FCV and MNV strains also have the ability to activate the COXs/PGE_2_ signaling pathway during their replication. To investigate this, we first evaluated the influence of FCV and MNV infections on COX gene and protein expressions. Expression levels of COX-2 mRNA and protein gradually increased from 1 h post-infection (hpi) (Fig 1A and 1C) in parallel with increased FCV viral RNA synthesis (Fig 1B), while COX-1 protein levels remained unchanged (Fig 1A and 1C). Similarly, the time-dependent increase in the expression of COX-2 mRNA and protein levels was observed in association with an increase in MNV mRNA level starting at 4 hpi (Fig 2A–2C). The expression level of COX-1 mRNA and protein was also unaffected during MNV infection (Fig 2A and 2C). Since PGE_2_ is one of the main products of the COX enzymes [29], we also assessed the expression levels of PGE_2_ during FCV and MNV infections. Concurrent with the increased COX-2 levels during MNV and FCV infections, the production of PGE_2_ was also elevated in a time-dependent manner starting from 1 hpi for FCV (Fig 1D) and from 4 hpi for MNV (Fig 2D).

**Fig 3:**
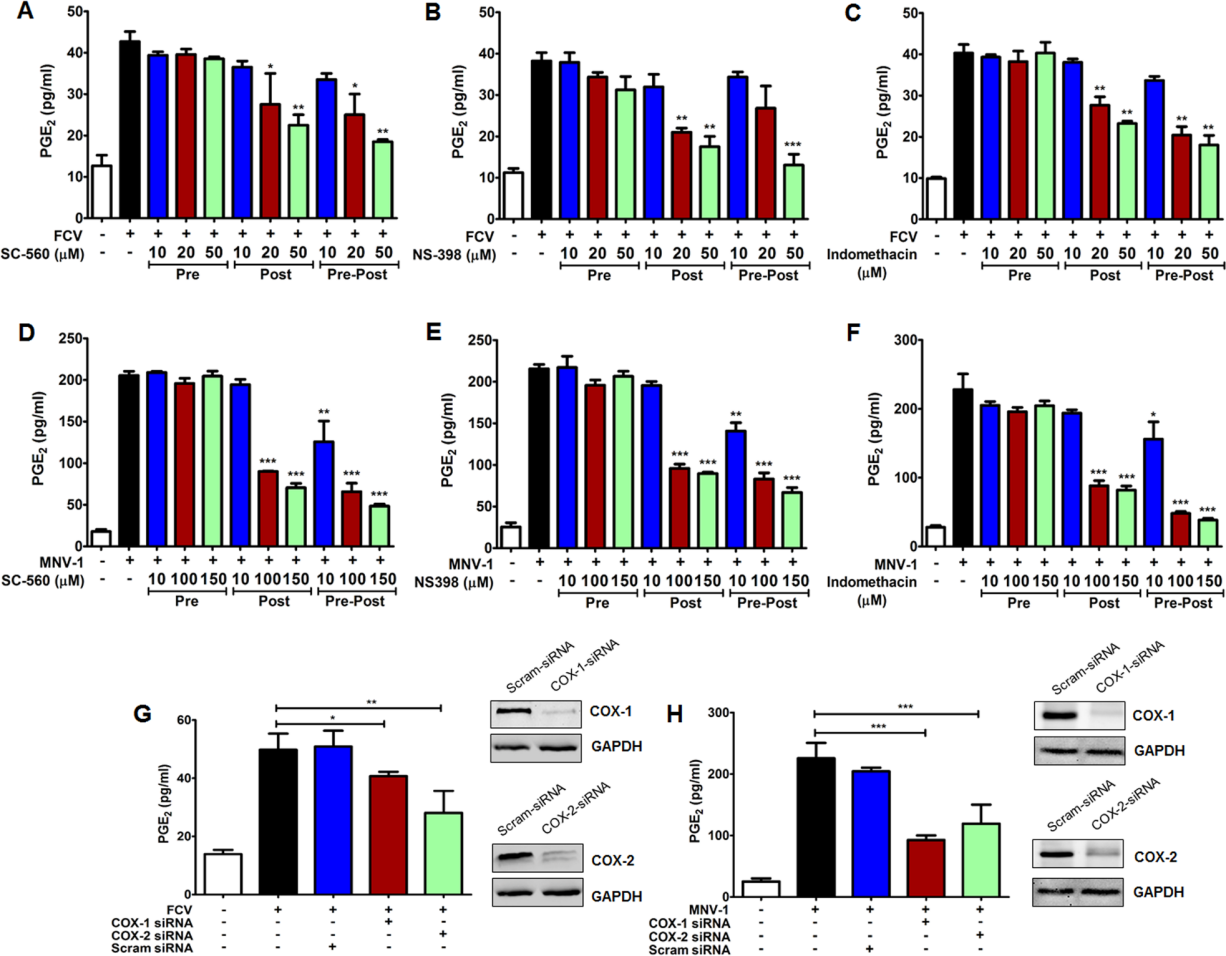
Influence of COX inhibitors on PGE_2_ production during feline calicivirus (FCV) and murine norovirus (MNV) replication. (A–F) CRFK and RAW264.7 cells were treated with a selective COX-1 inhibitor (SC-560), a selective COX-2 inhibitor (NS398), or a nonselective COX inhibitor (indomethacin) at the indicated time points. Cells pretreated with each inhibitor were washed to remove each inhibitor and then infected with FCV (MOI, 1 FFU/ml) or MNV (MOI, 1 TCID_50_/ml) strains (Pre). After virus adsorption, the inhibitor(s) were added in the maintenance media (Post), or before virus inoculation and maintained throughout the course of the infection (Pre-Post). The levels of PGE_2_ in the supernatants harvested at 4 hpi for FCV and 24 hpi for MNV were determined by ELISA. The PGE_2_ levels from virus-infected supernatants were compared between the mock- and drug-treated groups. (G and H) Confluent CRFK cells (G) and RAW264.7cells (H) were transfected with COX-1 and COX-2, or scrambled (Scram) siRNAs, and then infected with FCV (MOI, 1 FFU/cell) for 4 h or MNV (MOI, 1 TCID_50_/ml) for 24 h. Then, supernatants were collected and PGE_2_ levels were detected by ELISA. (Insets) CRFK cells (G) and RAW264.7 cells (H) transfected with COX-1, COX-2, or scrambled siRNAs were harvested and subjected to Western blot analyses. GAPDH was used as a loading control. The results are presented as means and standard errors of the mean of three independent experiments. Statistically analyses were performed using one-way analysis of variance. *p < 0.05; **p < 0.001; ***p < 0.0001.

To further confirm the correlation of increased PGE_2_ with the FCV- and MNV-mediated induction of COX-2, the influence of selective and nonselective COX inhibitors on PGE_2_ expression was investigated. Non-toxic doses were used for this study as determined by the MTT assay (S1 Fig). The selective COX-1 inhibitor SC-560, selective COX-2 inhibitor NS-398, and nonselective COX-1 and -2 inhibitor indomethacin hampered FCV mediated-PGE_2_ induction in a dose-dependent manner when added after the removal of the virus inoculum (post-treatment) or added before and after the virus adsorption step (pre-post-treatment) (Fig 3A–3C). Similarly, addition of COX inhibitors after removal of the MNV inoculum or during the entire course of the MNV infection reduced PGE_2_ levels (Fig 3D–3F). However, the pretreatment of cells and consequent removal of inhibitors before FCV or MNV infection did not lower PGE_2_ levels due to the reversible nature of the inhibitors (Fig 3A–3F).

To further corroborate the influence of COX genes in the production of PGE_2_ during FCV and MNV infections, the effects of siRNAs were also examined. RAW 264.7 and CRFK cells transfected with siRNAs against COX-1 or COX-2 genes showed a reduction in the expression levels of their corresponding target genes (Fig 3G and 3H). Transfection of siRNAs targeting COX-1 or COX-2 in RAW 264.7 and CRFK cells following FCV and MNV infections, respectively, significantly reduced the amount of PGE_2_ released (Fig 3G and 3H). These results confirmed that induction of COX-2, together with the basal expression of COX-1, accounted for the increase in PGE_2_ in FCV and MNV-infected cells.

### Inhibition of both COX enzymes reduce FCV and MNV replication

**Fig 4:**
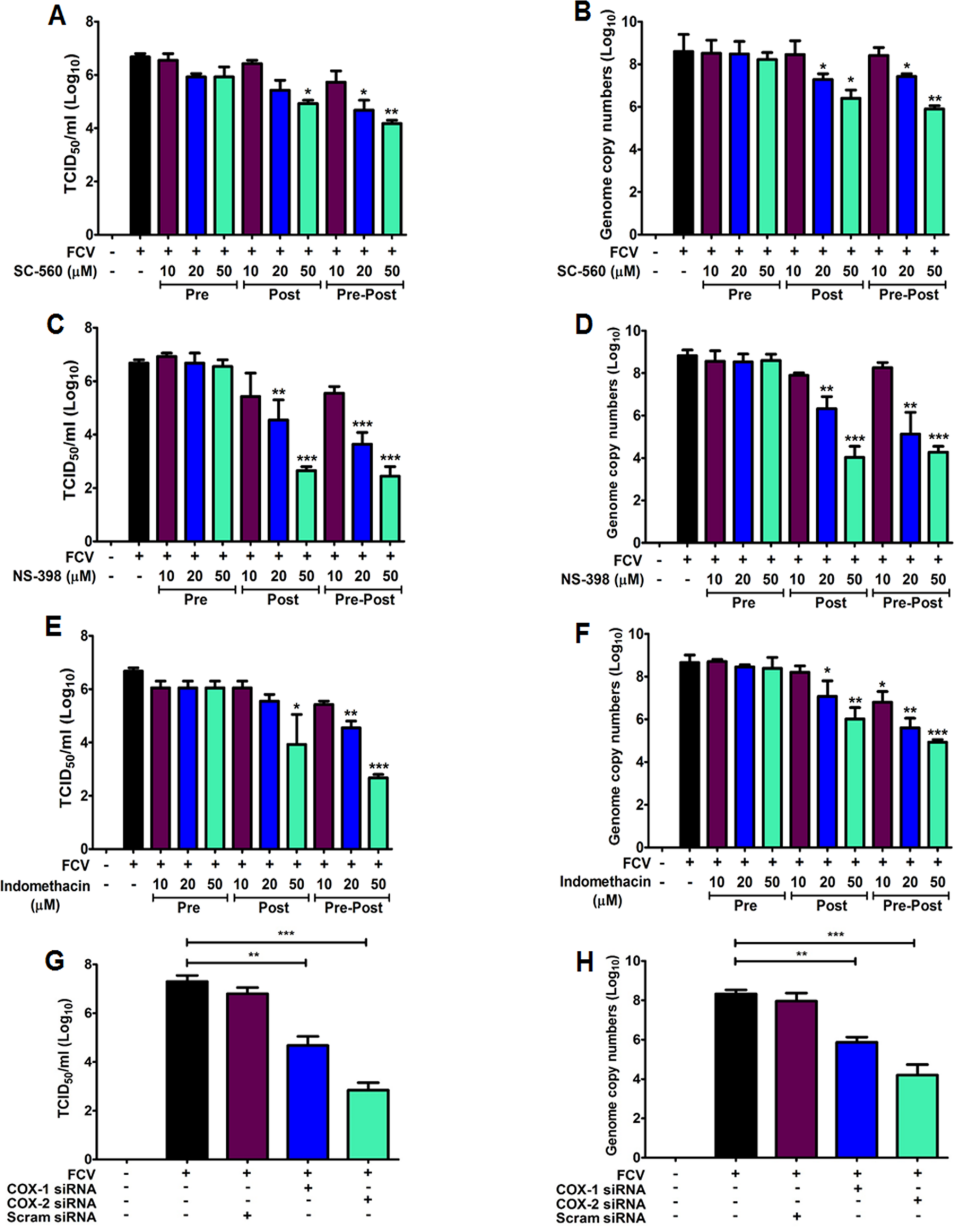
Inhibition of COX isoforms results in a dramatic reduction in feline calicivirus (FCV) progeny yield. (A–F) CRFK cells were pretreated (Pre), post-treated (Post), or pre-post-treated (Pre-Post) with non-cytotoxic doses of SC-560, NS-398, and indomethacin. At 8 h post-infection (hpi) with FCV (MOI, 1 FFU/cell), the viral titer and genome copy number were determined by TCID_50_/ml and quantitative real time PCR (qRT-PCR). The inhibitory effects of each drug on virus titer or genome copy number were compared between mock- and drug-treated groups. (G and H) COX-1, COX-2, or scrambled (Scram) siRNAs were transfected in CRFK cells before infection with FCV (MOI, 1 FFU/cell). The samples were harvested at 8 hpi, and then the viral titer and genome copy number were determined by TCID_50_/ml (G) and qRT-PCR (H) analyses. Three independent experiments were conducted and presented as means and standard errors of the mean. Statistical differences were determined using one-way analysis of variance. *p < 0.05; **p < 0.001; ***p < 0.0001.

**Fig 5:**
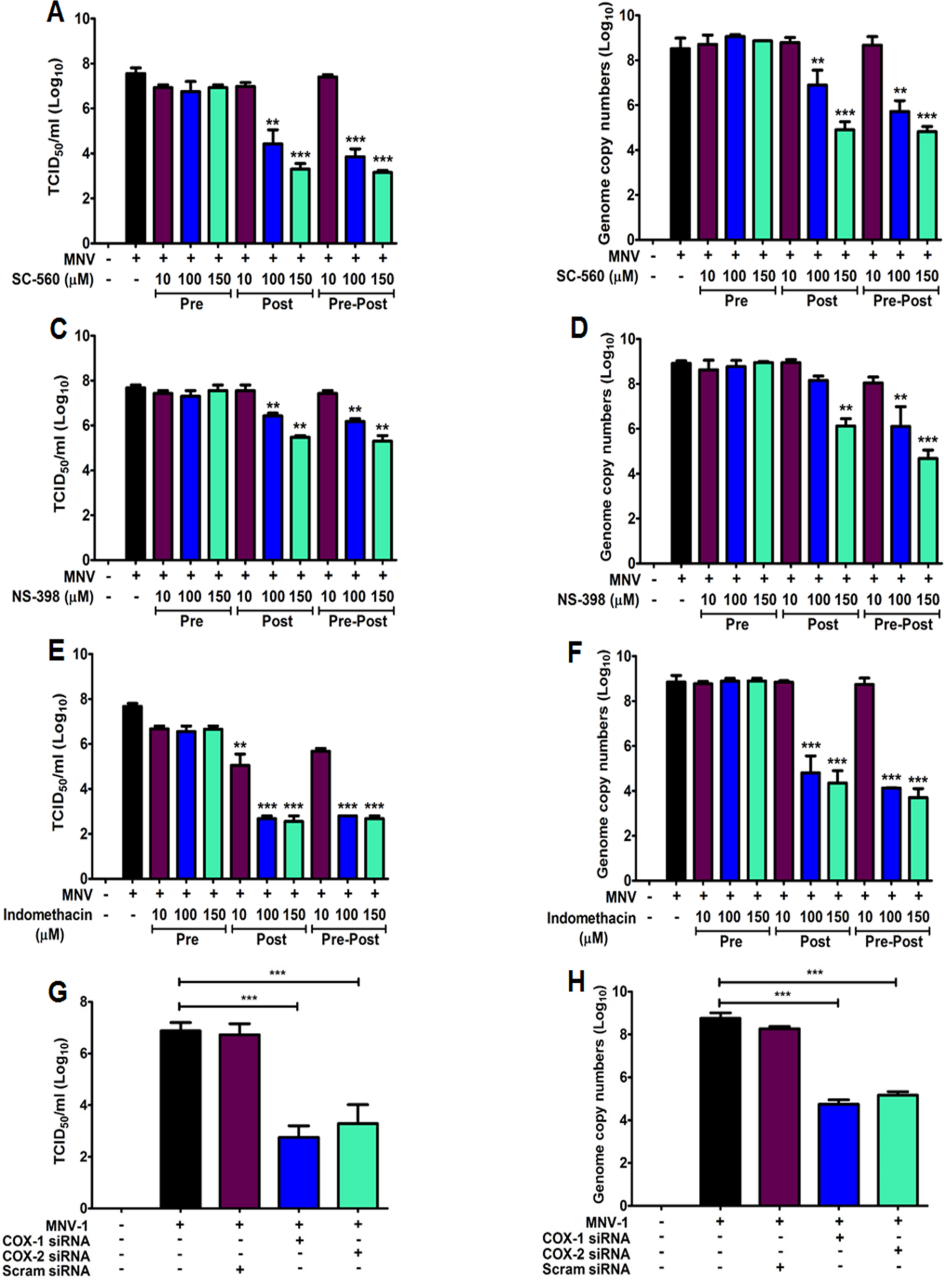
Murine norovirus (MNV) infection is hampered by COXs inhibitors. (A–F) RAW264.7 cells were pretreated (Pre), post-treated (Post), or pre-post-treated (Pre-Post) with non-cytotoxic doses of SC-560, NS-398, or indomethacin. At 24 h post-infection (hpi) with MNV (MOI, 1 TCID50/ml), viral titer and genome copy number were determined by TCID_50_/ml and quantitative real time PCR (qRT-PCR) analyses. The inhibitory effects of each drug on the viral titer or genome copy number were compared between mock- and drug-treated groups. (G and H) COX-1, COX-2, and scrambled (Scram) siRNAs were transfected in RAW264.7 cells before infection with MNV (MOI, 1 TCID_50_/ml). The samples were harvested at 24 hpi, and virus titer and genome copy number were determined by TCID_50_/ml (G) or qRT-PCR (H) analyses. The inhibitory effects of each siRNA on viral titer and genome copy number were compared between the mock- and each siRNA-transfected group. Three independent experiments were conducted and presented as means and standard errors of the mean. Statistical differences were determined using one-way analysis of variance. **p < 0.001; *** *p <* 0.0001.

To assess the effect of COX stimulation on the replications of FCV and MNV strains, we assessed the impact of COX inhibitors on FCV and MNV replication. RAW 267.4 and CRFK cells were pre-treated (Pre), post-treated (Post), or pre-post-treated (Pre-Post) with COX inhibitors, and viral replication was monitored at 8 hpi for FCV and 24 hpi for MNV by examining the viral titers and RNA levels. The selective COX-1 inhibitor SC-560 reduced the levels of infectious virus and RNA ~3 log_10_ for FCV (Fig 4A and 4B) and by ~4 log_10_ for MNV (Fig 5A and 5B) when added after infection (post-treatment) or maintained throughout the virus infection (pre-post-treatment). Conversely, the addition of NS-398 (selective COX-2 inhibitor) following the removal of the virus inoculum (post-treatment) or during the entire progression of infection (pre-post-treatment) caused a more significant reduction in FCV (~4.5 log_10_) in both infectious virus and viral RNA (Fig 4C and 4D), while MNV infectious viral titer and genome copy numbers were reduced ~3 log_10_ (Fig 5C and 5D). Indomethacin, a nonselective COX inhibitor, showed more significant effects on MNV infection, leading to a ~5 log_10_ reduction in virus yield and RNA synthesis (Fig 5E and 5F), while FCV infection elicited a ~3 log_10_ decrease in infectious viral titer and genome copy numbers (Fig 4E and 4F) when added after virus adsorption or maintained throughout the course of the infection. There was no significant effect on FCV or MNV replication in cells pretreated with COX inhibitors (Figs 4A–4F and 5A–5F). We also assessed the effect of COX-specific siRNAs on FCV and MNV replication. FCV genome copy numbers and titers were more strongly reduced (~4 log_10_) in cells transfected with siRNA against COX-2 than that in cells transfected with COX-1 siRNA (~2 log_10_) (Fig 4G and 4H). Transfection of COX-1- or COX-2-specific siRNAs resulted in similar substantial reduction in MNV replication, causing a ~4 log_10_ decrease in infectious viral titer and RNA levels (Fig 5G and 5H).

**Fig 6:**
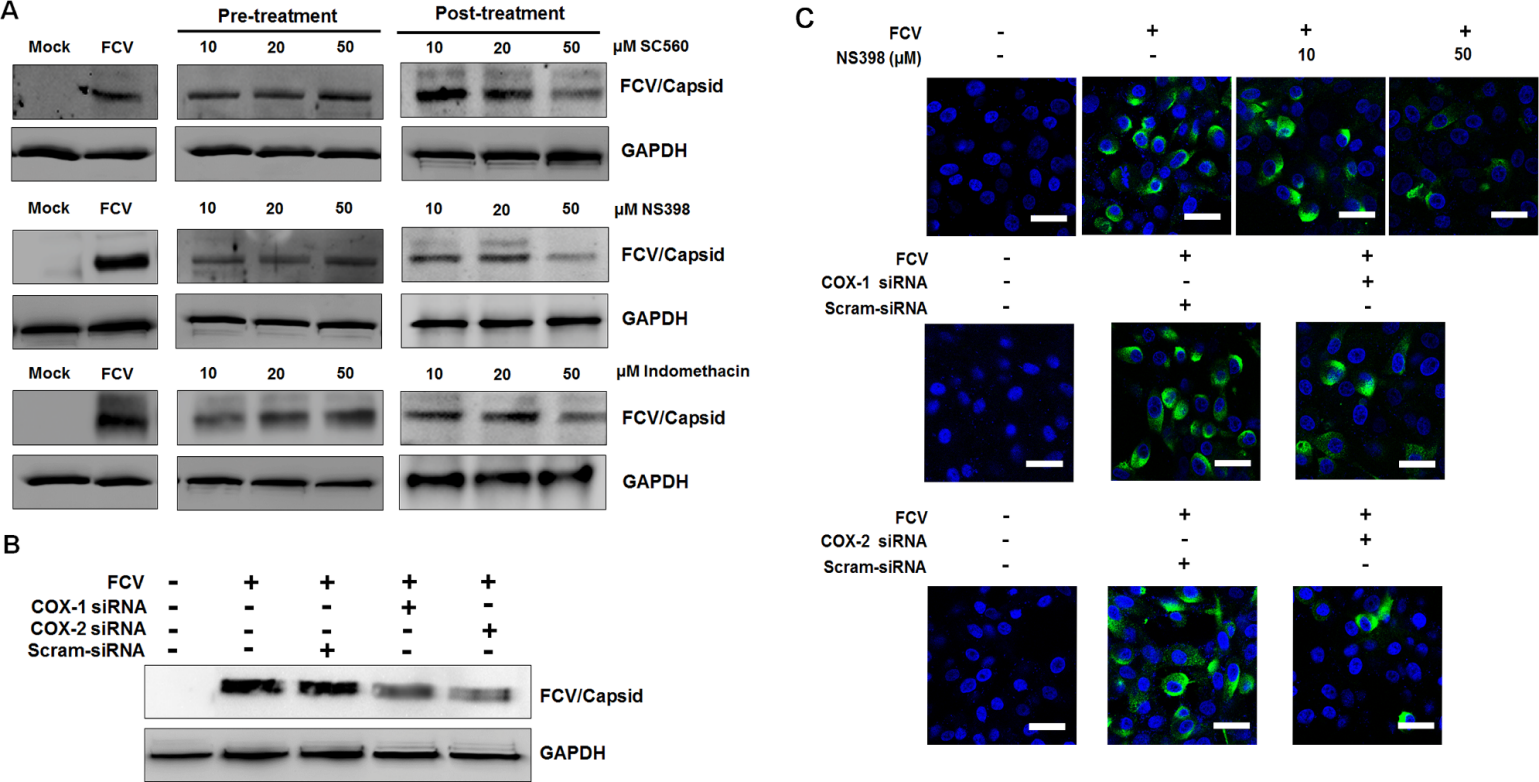
Effects of COX inhibitors or siRNAs on feline calicivirus (FCV) replication. (A) CRFK cells were pretreated or post-treated with noncytotoxic doses of SC-560, NS398, and indomethacin. At 8 h post-infection (hpi) with FCV (MOI, 1 FFU/ml), the levels of viral capsid protein were determined by Western blot analysis. GAPDH was used as a loading control. (B) CRFK cells transfected with COX-1, COX-2, or scrambled (Scram) siRNAs were incubated with FCV (MOI, 1 FFU/ml) for 8 h. Harvested samples were processed for Western blot analysis to detect FCV capsid protein. GAPDH was used as a loading control. (C) CRFK cells were treated with conditions described immediately above, and the effect of the COX-2 inhibitor NS398, or COX-1, COX-2, and scrambled (Scram) siRNAs on viral capsid protein production was determined by confocal microscopy. Bar = 20 μM.

**Fig 7:**
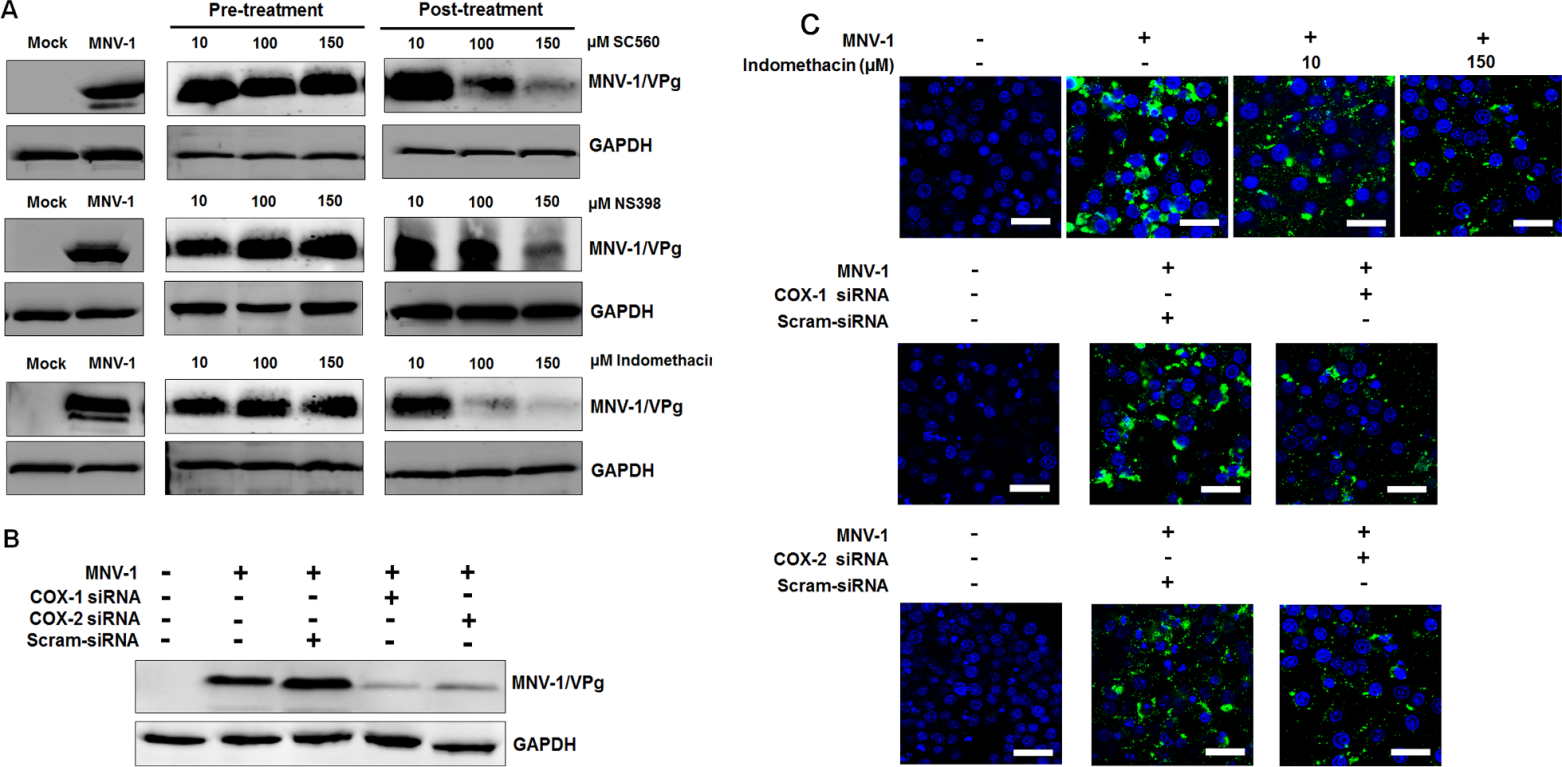
Inhibition of COX isoforms on murine norovirus (MNV) infection leads to decreased MNV replication. (A) Cultured RAW 264.7 cells were pretreated or post-treated with noncytotoxic doses of SC-560, NS398, or indomethacin. Samples were harvested at 24 h post-infection (hpi), and the levels of viral protein VPg were determined by Western blot analysis. GAPDH was used as a loading control. (B) RAW264.7 cells were transfected with COX-1, COX-2, or scrambled (Scram) siRNAs and incubated with MNV (MOI, 1 TCID_50_/ml) for 24 h. Western blot analysis was performed to detect MNV VPg protein. GAPDH was used as a loading control. (C) RAW 264.7 cells were mock- or post-treated with a selective COX-2 inhibitor indomethacin, or non-transfected or transfected with COX-1, COX-2, or Scram siRNAs. The cells were then incubated with MNV (MOI, 1 TCID_50_/ml) for 24 h, and the effect of the drugs or COX-1, COX-2, or Scram siRNAs on the expression level of viral VPg protein was determined by confocal microscopy. Bar = 20 μM.

To further assess the effect of COX inhibitors on FCV and MNV replication, we also analyzed viral protein levels in the pre- and post-treatment groups. Consistent with the above results, a reduced production of FCV capsid protein was observed with the post-treatment of COX inhibitors in a dose-dependent manner, in which NS398 showed stronger anti-FCV effects against other inhibitors (Fig 6A). The expression level of MNV VPg protein decreased due to the COX inhibitors in a dose-dependent manner, particularly with the post-treatment of indomethacin (Fig 7A). Transfection of COX-1 and COX-2 siRNAs resulted in lower protein induction in both FCV- and MNV-infected cells (Figs 6B and 7B). Furthermore, treatment of CRFK cells with NS-398 or transfection with COX-1 and COX-2 specific siRNAs caused a significant decreased in the number of FCV antigen-positive cells (Fig 6C). Similarly, a reduced number of MNV antigen-positive cells was also observed in RAW264.7 cells treated with NS-398 or transfected with COX-1 or COX-2 specific siRNAs (Fig 7C). Together, these results suggested that both COX-1 and COX-2 augment FCV and MNV replication possibly in relation with the increased levels of PGE_2_.

### Addition of PGE_2_ restores the inhibitor effect of COXs on FCV and MNV replication

**Fig 8:**
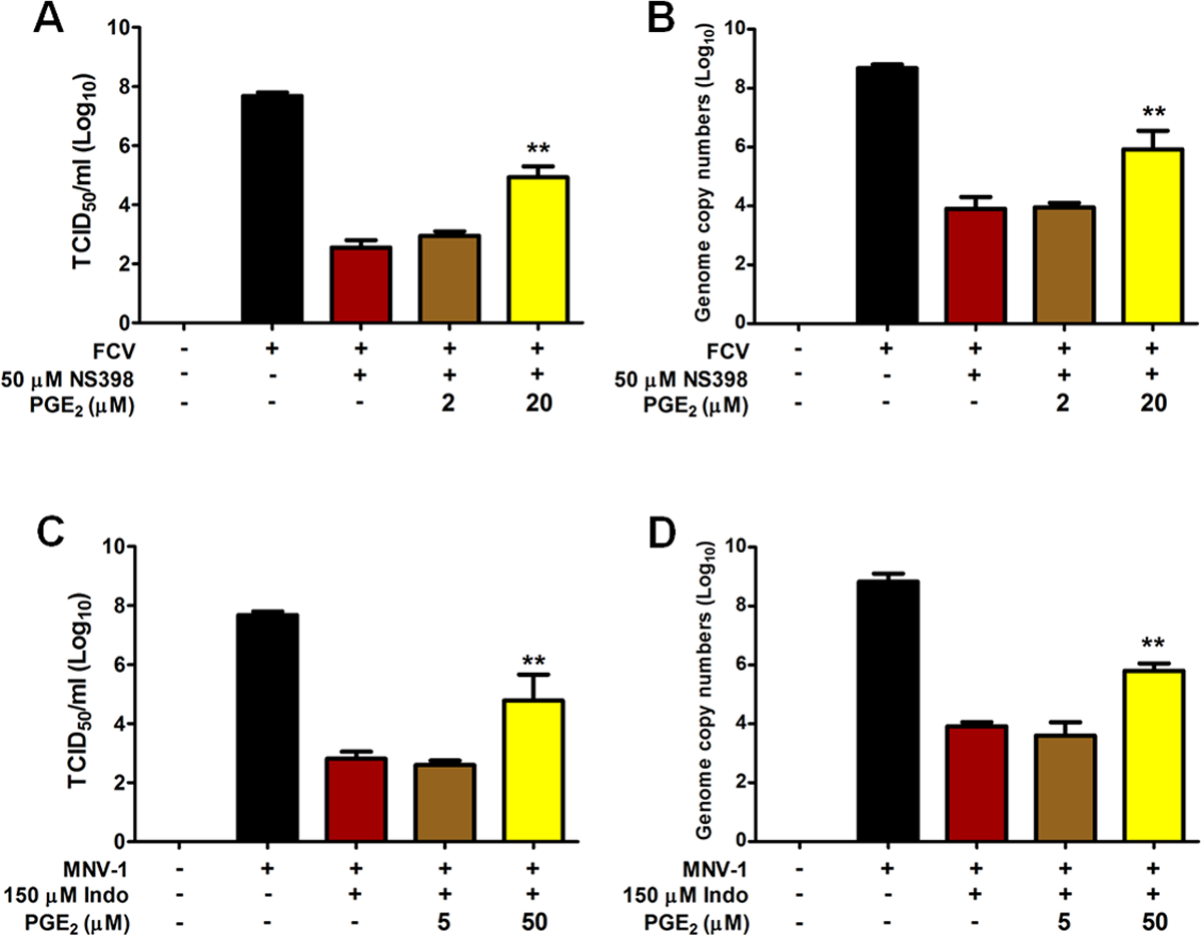
Addition of exogenous prostaglandin E2 can compensate for COX-induced inhibition of feline calicivirus (FCV) and murine norovirus (MNV) replications. (A–D) CRFK cells and RAW264.7 were infected with FCV (MOI, 1 FFU/ml) and MNV (MOI, 1 TCID_50_/ml), respectively, and then treated with noncytotoxic doses of indomethacin and NS-398 for MNV and FCV, respectively. Two doses of exogenous PGE_2_, 5 or 50 μM for MNV, and 2 μM or 20 μM for FCV, were then added in the maintenance media, and samples were harvested at 8 h post-infection (hpi) for FCV and 24 hpi for MNV. Viral titer and viral genome copy numbers were determined by TCID_50_ and quantitative real time PCR analyses and compared between mock- and drug-treated groups. The data presented are means and standard errors of the mean from three different experiments. Statistical analysis was performed using one-way analysis of variance. ** *p <* 0.001.

To further study the proviral effect of COXs/PGE_2_ induction during FCV and MNV infections, we assessed whether the addition of exogenous PGE_2_ restores the inhibitory effect of COX inhibitors on viral replication. The selective COX-2 inhibitor NS-398 was used for FCV, while the nonselective inhibitor indomethacin was used for MNV, both of which showed strong inhibitory effect compared with other COX inhibitors. Non-cytotoxic doses of COX-inhibitors in combination with exogenous PGE_2_ were used in this experiment. The addition of PGE_2_ lead to the restoration of both FCV and MNV progeny production and viral RNA levels in a dose-dependent manner (Fig 8A–8D). Collectively, these results confirmed that PGE_2_, the final product of both COX enzymes, mediated the proviral effects on FCV and MNV replication.

## Discussion

Depending on the viruses, the COXs/PGE_2_ signaling pathway was used differently: COX-1/PGE_2_ for PRRS arterivirus [35], COX-2/PGE_2_ for many viruses [36, 37, 39–42, 46–49, 50–53], or both for pseudorabies herpesvirus [54]. In the present study, both FCV and MNV strongly activated the COX-2/PGE_2_ pathway from an early replication time point. These results are different from our previous finding where the PSaV strain Cowden, belonging to the same *Caliciviridae* family, induced late activation of the COX-2/PGE_2_ signaling pathway [34]. This suggested that the activation time of the COX-2/PGE_2_ signaling pathway was different even in the same *Caliciviridae* family. In addition, the PSaV Cowden strain transiently activated COX-1/PGE_2_ signaling during the later stage of infection [34]. Interestingly, no increases in the COX-1 mRNA and protein levels were observed during the entire FCV and MNV infection. However, silencing of COX-1 by specific siRNAs as well as the inhibition of COX-1 by the specific inhibitor SC-560 decreased the production of PGE_2_ and replication of both viruses. Although the detailed mechanism of these results is unknown, it is presumed that the inhibition of the basal expression level of COX-1 by its specific inhibitor and siRNA suppressed PGE_2_ production and thereby the replication of both viruses. Based on these results, it could be concluded that unlike the PSaV-induced late activation of both COX-1 and COX-2/PGE_2_ signaling pathways, both FCV and MNV activate only COX-2/PGE_2_ signaling pathway from early replication, but COX-1 might be an important factor for the successful replication of FCV and MNV.

In relation to the virus replication, the COX/PGE_2_ signaling pathway can have proviral [36, 37, 41, 42, 45, 50–52, 54, 55, 57, 58, 69], or antiviral [33, 43–45, 55, 56, 59] effects. In the present study, the inhibition or silencing of COX-2 reduced replication of FCV and MNV. Moreover, reduced viral replication by COX inhibitors was restored by addition of PGE_2_. Similar results have been recently reported in a PSaV infection study [34]. These data indicate that like PSaV [34], FCV- and MNV-induced activation of the COX-2/PGE_2_ signaling pathway acts as a proviral mechanism for the replication of FCV and MNV.

Nonsteroidal anti-inflammatory drugs (NSAIDs) targeting COX enzymes are one of the most widely prescribed drugs due to their analgesic effects and their potent anti-inflammatory and anti-pyretic properties in the western world [26, 70]. NSAIDs have been reported as potential therapeutic drugs for some viral infection. For example, pharmacological use of a COX-2 inhibitor in combination with neuraminidase inhibitors enhanced the survival of mice infected with H5N1 influenza [71]. The selective COX-2 inhibitor NS-398 was shown to protect mice from succumbing to dengue virus-2 infection [41]. In this study, we determined the potency of different COX inhibitors against FCV and MNV infections. In the FCV infection, the COX-2 selective inhibitor NS398 exerted ~4 log_10_ suppression in the FCV titer compared to other inhibitors. Among the COX inhibitors tested against MNV, indomethacin, a nonselective COX inhibitor, showed stronger anti-MNV effects than other inhibitors, leading to a ~5 log_10_ decrease in MNV titer. These results suggest that COX inhibitors are potential candidates for the treatment of FCV infections, which are particularly serious problems for cats [12, 14]. Future studies are needed to determine whether *in vivo* treatments with COX inhibitors are effective in treating FCV and MNV infections.

Nitric oxide (NO) has been shown to exert antiviral effects during virus infections [72–76]. It has also been shown that the COX/PGE_2_ signaling pathway also regulate inducible nitric oxide synthase, the enzyme responsible for the production of NO [77]. *In vitro* and *in vivo* vesicular stomatitis virus infection has been shown to activate the COX-2/PGE_2_ signaling pathway, which in turn restrict NO production [37, 46]. In the same way, we demonstrated that PSaV-induced activation of COX-2/PGE_2_ signaling pathway inhibited NO production [34] to favor PSaV replication. A similar response is expected in FCV and MNV infections, which is the ongoing goal of future studies.

In a few cases, viral proteins could activate COX/PGE_2_ signaling, either directly or indirectly as a transcriptional transactivator of COX-2 gene expression [42, 78–80]. For example, the HCV proteins such as NS3 and NS5A are known to enhance the COX-2/PGE_2_ pathway by activating multiple signaling pathways [81, 82]. In severe acute respiratory syndrome coronavirus infection, viral nucleocapsid protein activates COX-2/PGE_2_ pathway through direct binding to regulatory elements for NF-κB and CCAAT/enhancer binding protein [80]. In our previous report, PSaV VPg and ProPol proteins significantly enhanced the expression of COX-2/PGE_2_ signaling pathway [34]. Likewise, some FCV and MNV proteins are anticipated to activate the COX-2/PGE_2_ signaling pathway, the mechanism of which will be investigated in a future study.

In conclusion, our results denote a crucial role for the COX-2/PGE_2_ signaling pathway in the successful replication of FCV and MNV by creating a cellular environment suitable for efficient growth for both viruses. Furthermore, our results indicate that NSAIDs, pharmacological inhibitors against COX-1 and -2 enzymes, can be used as therapeutic drugs for FCV, MNV, and other members such as human noroviruses.

## Supporting information

S1 Fig Cytotoxic assays for different chemicals used in the study. RAW264.7 9 (A–D) and CRFK (E–H) cells were treated with different concentrations of the selective COX-1 inhibitor SC-560, selective COX-2 inhibitor NS-398, nonselective COX inhibitor indomethacin (Indo), and exogenous prostaglandin E2 (PGE_2_). A MTT assay was performed to determine the noncytotoxic doses to use in this study.
(TIF)
