# Supplementary figures and images for: Feline calicivirus- and murine norovirus-induced COX-2/PGE2 signaling pathway has proviral effects
Source: PLoS One. 2018 Jul 18;13(7):e0200726. doi: 10.1371/journal.pone.0200726 (PMC6051663; doi:10.1371/journal.pone.0200726)

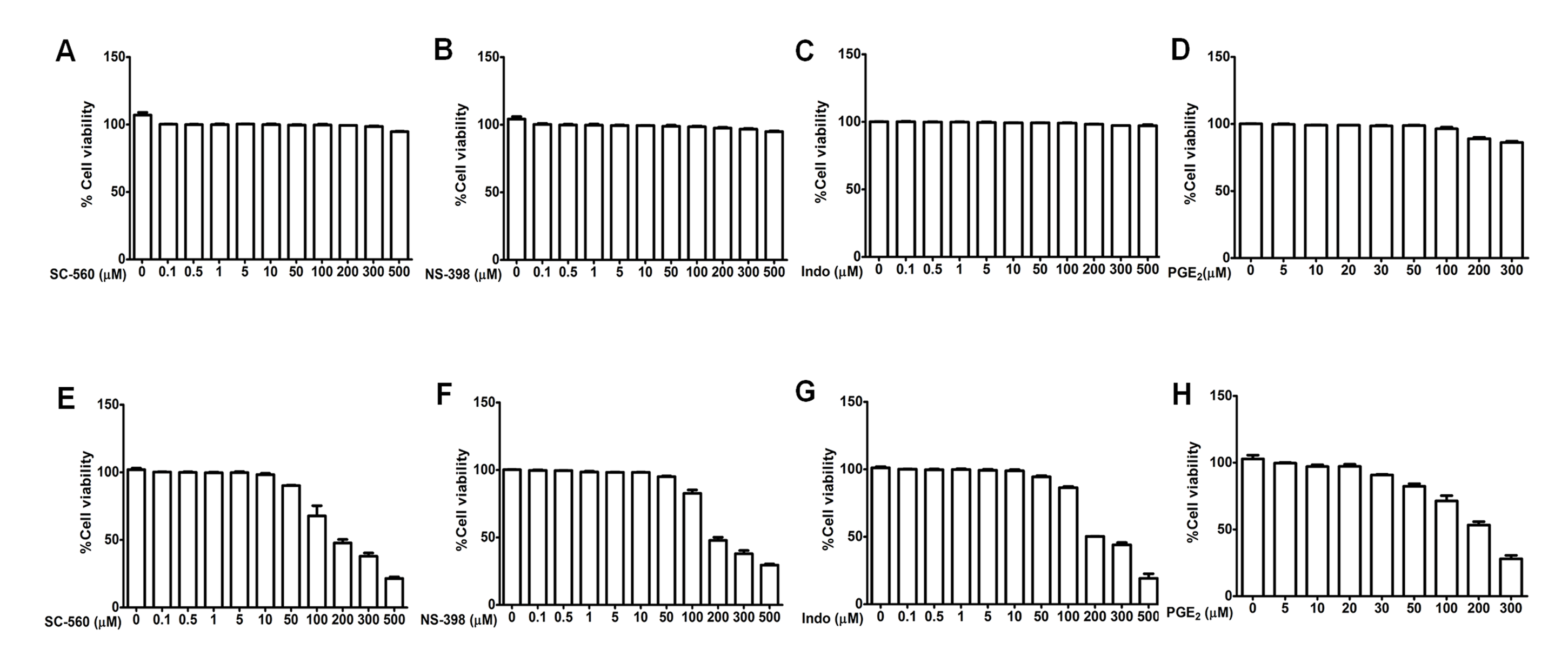

Supplement: S1 Fig — RAW264.7 9 (A–D) and CRFK (E–H) cells were treated with different concentrations of the selective COX-1 inhibitor SC-560, selective COX-2 inhibitor NS-398, nonselective COX inhibitor indomethacin (Indo), and exogenous prostaglandin E2 (PGE2). A MTT assay was performed to determine the noncytotoxic doses to use in this study. (TIF) [file pone.0200726.s001.tif]
